# Early weight gain influences duration of breast feeding: prospective cohort study

**DOI:** 10.1136/archdischild-2022-323999

**Published:** 2022-07-15

**Authors:** Laurentya Olga, Janna A van Diepen, Gabriele Gross, David B Dunger, Ken K Ong

**Affiliations:** 1 Department of Paediatrics, University of Cambridge, Cambridge, UK; 2 Medical and Scientific Affairs, Reckitt / Mead Johnson Nutrition Institute, Nijmegen, The Netherlands; 3 Wellcome Trust-MRC Institute of Metabolic Science, University of Cambridge, Cambridge, UK; 4 MRC Epidemiology Unit, University of Cambridge, Cambridge, UK

**Keywords:** epidemiology, paediatrics, growth, child health

## Abstract

**Objective:**

While several studies have shown that milk formula feeding is associated with faster infant weight gain compared with exclusively breast feeding (EBF), we explored the possible reverse association that infant weight gain influences the duration of EBF.

**Design:**

Prospective birth cohort study (Cambridge Baby Growth Breastfeeding Study) born 2015–2018.

**Setting:**

Cambridge, UK.

**Participants:**

Full-term, singleton, normal birthweight infants who received EBF for 2–5 completed weeks (n=54), 6–11 weeks (n=14) or 12 or more weeks (n=80).

**Intervention:**

Weight gain from birth to 2 and 6 weeks.

**Main outcome and measure:**

Duration of EBF.

**Results:**

Faster infant weight gain during EBF predicted longer duration of EBF. Among all 148 infants, each +1 unit gain in weight SD score (SDS) between birth and 2 weeks (while all infants received EBF) reduced the likelihood of stopping EBF between 2 and 5 weeks by ~70% (OR 0.32; 95% CI 0.12 to 0.77; adjusted for sex, gestational age at birth, birth weight and mother’s age, prepregnancy BMI and education). Similarly, among infants EBF for 6 or more weeks (n=94), each +1 unit gain in weight SDS between birth and 6 weeks reduced the likelihood of stopping EBF between 6 and 11 weeks by ~80% (OR 0.18; 95% CI 0.05 to 0.63).

**Conclusions:**

Slower early infant weight gain was consistently associated with subsequent earlier discontinuation of EBF. We conjecture that broader recognition of the wide range of normal infant growth might encourage parents to not stop EBF earlier than they intended.

## Abstract (precis)

In this longitudinal cohort study of term, singleton, normal birthweight, initially exclusively breastfed infants, slower weight gain while exclusively breast feeding was associated with higher likelihood of introduction of infant milk formula in subsequent follow-up periods, between 2 and 11 weeks.

**WHAT IS ALREADY KNOWN ON THIS TOPIC**

- Several studies show that formula feeding is associated with faster infant weight gain compared with exclusively breast feeding (EBF).
- Conversely, growth charts based on breastfed infants describe heavier infant weights in the first 2–3 months compared with charts based on mostly formula-fed infants.

**WHAT THIS STUDY ADDS**

- In this cohort of initially EBF infants, slower weight gain while EBF was consistently associated with higher likelihood of introduction of infant milk formula during the subsequent follow-up periods, between ages 2 and 11 weeks.

**HOW THIS STUDY MIGHT AFFECT RESEARCH, PRACTICE OR POLICY**

- We conjecture that broader recognition of the wide range of normal infant growth might encourage parents to not stop EBF earlier than they intended.
- Current growth references, based on infants who were exclusively or predominantly breast fed for at least 4 months, may overestimate early weight gain in the wider population of infants who start EBF.

## Introduction

Several studies have reported that formula-fed (FF) infants grow faster than those who are exclusively breast fed (EBF).^1–3^ This early growth difference has implications for long-term risks for overweight and obesity^4^ and formed the rationale for the development and widespread adoption of growth standards based on EBF or predominantly breastfed infants.^5^

However, not all data are consistent with this notion. First, in some resource-poor settings, FF infants show slower weight gain than EBF infants, plausibly due to the relatively high cost of infant formula and the high absolute risks of gastrointestinal and respiratory infections associated with FF in those settings.^6^ Second, even in affluent settings, some data show that EBF infants are initially heavier than FF infants during the first 2–3 months. A notable example is the comparison of average growth between the WHO 2006 Growth Standards (based on EBF or predominantly breastfed infants) versus the US Centers for Disease Control (CDC) reference (majority FF). The median weight in the WHO standards was above the CDC median during the ﬁrst half of infancy—this early weight advantage of the WHO median peaked at age 2 months, reduced to zero at ~6 months, and then continued to decline steadily after 6 months.^7^ A similar pattern was seen when comparing the WHO standards with the British 1990 growth reference (majority FF), with an initial weight advantage only in the first 3–4 months.^8^ Comparison of early infant weight velocities, derived from these three growth references, clearly demonstrates the faster weight gain during the first 2 months inferred by the WHO standards (online supplemental figure 1).

10.1136/archdischild-2022-323999.supp1 Supplementary data

Such age-graded differences in growth between FF and breastfed infants are at odds with the possible mechanisms. Consistently, FF infants have higher energy and protein intakes than breastfed infants.^1 3^ The posited reasons for this are: infant formula is more energy dense than breastmilk; infant formula has higher protein contents or other composition differences to breastmilk and the process of bottle-feeding may more easily allow overfeeding, whether intentional or unintentional,^9^ whereas the mechanics of breast feeding allow infants to better learn to self-regulate.^1 10^ Therefore, there are several mechanisms to explain why FF might grow faster than EBF infants, but not (at least in affluent settings) why FF infants should grow more slowly than EBF infants. This suggests the possibility of an artefactual difference in early infancy growth patterns in some studies due to selection bias. We hypothesised that slower initial weight gain in EBF infants might lead to earlier stopping of EBF (ie, ‘reverse causality’).

## Methods

### Study population

The Cambridge Baby Growth Breastfeeding Study (CBGS-BF) is an observational cohort study that recruited mother-infant pairs at birth from a single centre, the Rosie Maternity Hospital, Cambridge, UK.^11^ Inclusion criteria were: healthy term vaginally delivered singletons without low birth weight (> −1.5 SD score (SDS) by the British 1990 growth reference) and with intention to exclusive breast feeding (EBF) from birth to age 6 weeks or more. Exclusion criteria included mother’s age <16 years or unable to provide informed consent. To allow standardisation for the microbiome aspect of this study (not included here), further exclusion criteria were: maternal prepregnancy body mass index (BMI) >30 kg/m^2^), any significant maternal illness or pregnancy comorbidity, use of antibiotics or steroids in 30 days before delivery and regular consumption of probiotics. In total, 150 mother-infant pairs were recruited of whom 148 infants received EBF for at least 2 weeks and 94 were EBF for at least 6 weeks and were therefore eligible for further follow-up. Study size was determined by feasibility to assess breastmilk intake volumes using a deuterium-labelled water technique, which were used to inform other study aims.^11^

### Measurements

Birth weight at delivery was taken from routine medical records. Other birth measurements (table 1) were conducted by the research team in the first 8 days of life. At ages 2 weeks, 6 weeks, 12 weeks (3 months), 6 months and 12 months, infant weight and length were also measured by the research team. A Seca 757 electronic baby scale (Seca, Hamburg, Germany) was used to measure infant weight to the nearest 1 g. Infants were weighed before feeding, naked without diapers. Breastfeeding status was recorded at birth and at each study visit using a structured questionnaire.

**Table 1:** Characteristics of the study population, stratified by duration of exclusive breast feeding

Characteristics	Duration of exclusive breast feeding		
	2–5 weeks (n=54)	6–11 weeks (n=14)	12+ weeks (n=80)
Maternal			
Age at delivery (years)	32.7 (4.1)	32.8 (3.4)	33.4 (4.9)
Prepregnancy BMI (kg/m^2^)	23.2 (3.6)	21.0 (2.8)	22.5 (2.5)
Height (cm)	166.6 (6.3)	166.8 (6.8)	166.7 (6.2)
Primiparous (%)	24%	36%	38%
White European (%)	92%	93%	91%
University education (%)	69%	57%	69%
Infant at birth			
Male sex (%)	59%	50%	63%
Gestational age (weeks)	40.2 (1.0)	40.6 (1.1)	40.2 (1.0)
Weight SDS	0.08 (0.76)	0.31 (0.68)	0.08 (0.76)
Length SDS	−0.26 (0.82)	−0.24 (0.60)	−0.2 (0.76)
BMI SDS	0.08 (0.99)	0.38 (0.99)	0.03 (0.84)

Values are mean (SD) or %.

There was no statistical difference between groups in any characteristic by analysis of Variance.

BMI, body mass index; SDS, SD score.

### Statistics

Age-adjusted and sex-adjusted SDS for weight were calculated according to the current UK guideline (ie, British 1990 growth reference at birth; subsequently WHO International Growth Standard).^8^ Growth was calculated as the difference in weight SDS (delta weight SDS) follow-up visits. Follow-up to age 12 months was complete in the 94 infants who were still EBF at 6 weeks. Differences in baseline characteristics and growth outcomes between groups categorised by EBF duration were tested by analysis of variance. Among the 148 infants who received EBF at 2 weeks, logistic regression was used to test the relationship of delta weight SDS between birth and 2 weeks on risk of stopping EBF at 2–5 completed weeks, including the following covariables: infant sex, gestational age at birth, weight SDS at birth and mother’s age, prepregnancy BMI and education. Among the 94 infants who were still EBF at 6 weeks, a similar logistic regression model was also used to test the relationship of delta weight SDS between birth and 6 weeks on risk of stopping EBF at 6–11 completed weeks.

## Results

Of the 148 infants who received EBF for at least 2 weeks, n=54 stopped EBF between 2 and 5 completed weeks, n=14 stopped EBF between 6 and 11 weeks and the remaining n=80 continued EBF for 12 or more weeks. All infants who stopped EBF at 2–5 or 6–11 weeks started on infant formula (ie, none started on solid food when stopping EBF). These groups did not differ in any maternal or birth characteristic (table 1).

**Figure 1:**
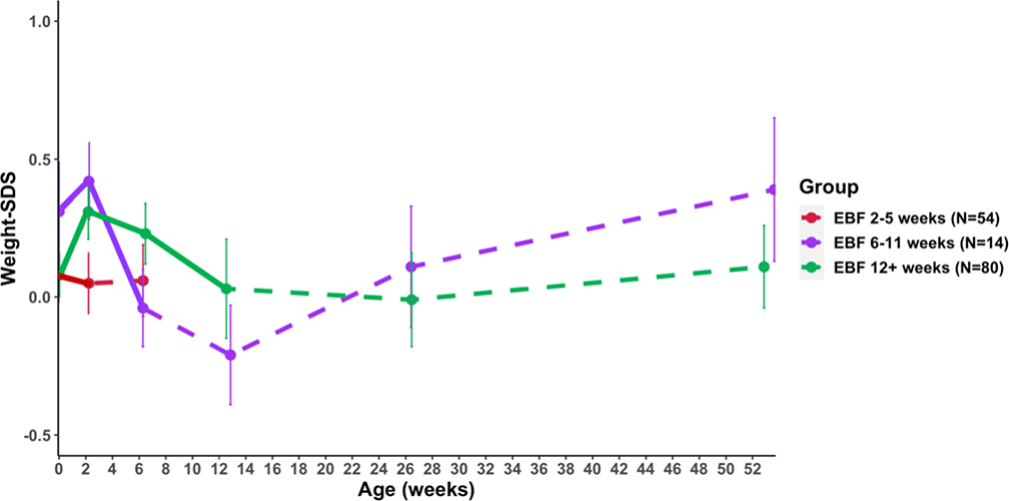
Infant weight SD scores (SDS) from birth to 52 weeks, stratified by exclusive breastfeeding (EBF) duration. Points and error bars indicate means and SEs. The solid lines indicate the EBF period in each group.

Prior to 2 weeks, while all infants were EBF (n=148), weight gain was fastest in infants who received EBF for 12 or more weeks (n=80) compared with infants who stopped EBF between 2 and 5 weeks (n=54; p=0.008; table 2 and figure 1). Similarly, among those infants who received EBF for at least 6 weeks (n=94), weight gain up to 6 weeks was faster among infants who continued EBF for 12 or more weeks (n=80) than infants who stopped EBF between 6 and 11 weeks (n=14; p=0.02). Conversely, after age 12 weeks, weight gain was faster in infants who stopped EBF between 6 and 11 weeks (n=14) compared with infants who continued EBF for 12 or more weeks (n=80; p=0.02).

**Table 2:** Delta weight SDS during infancy, stratified by duration of EBF

Period of weight gain (week)	Delta weight SDS by duration of EBF			2–5 weeks vs 12+ weeks	6–11 weeks vs 12+ weeks
	2–5 weeks (n=54)	6–11 weeks (n=14)	12+ weeks (n=80)		
0–2	−0.04 (0.08)	0.10 (0.15)	0.23 (0.06)	P=0.008	P=0.3
2–6	0.07 (0.13)	−0.45 (0.19)	−0.08 (0.11)	P=0.09	P=0.02
6–12	NA	−0.17 (0.11)	−0.2 (0.04)	NA	P=0.97
12–52	NA	0.61 (0.11)	0.08 (0.08)	NA	P=0.02

Values are mean (SE).

Tests of differences between groups were adjusted for infant sex, gestational age at birth, weight SDS at birth and mother’s age, prepregnancy BMI and education.

BMI, body mass index; EBF, exclusive breast feeding (duration in completed whole weeks); NA, not available; SDS, SD score.

Among all 148 infants, each +1 unit gain in weight SDS between birth and 2 weeks (while all infants received EBF) reduced the likelihood of stopping EBF between 2 and 5 weeks by ~70% (OR 0.32; 95% CI 0.12 to 0.77; adjusted for sex, gestational age at birth, birth weight and mother’s age, prepregnancy BMI and education). Similarly, among infants EBF for 6 or more weeks (n=94), each +1 unit gain in weight SDS between birth and 6 weeks reduced the likelihood of stopping EBF between 6 and 11 weeks by ~80% (adjusted OR 0.18; 95% CI 0.05 to 0.63). Infants who continued on EBF for 12–25 weeks (n=52) or 26 or more weeks (n=28) showed similar growth patterns from birth to age 52 weeks (online supplemental figure 2).

10.1136/archdischild-2022-323999.supp2 Supplementary data

## Discussion

In this observational study, slower weight gain while infants were receiving EBF was associated with subsequent earlier discontinuation of EBF. Similar ORs were seen for weight gain from birth to 2 weeks and birth to 6 weeks on the likelihood of subsequently stopping EBF at 2–5 weeks and 6–11 weeks, respectively. While most other studies aim to understand the question ‘how does infant feeding influence growth?’, these findings indicate ‘reverse causality’; that is, early infant growth rate influences feeding choice. Thereafter, beyond age 12 weeks, infants who changed from EBF to infant formula or mixed feeding gained weight more rapidly than infants who continued EBF.

Reverse causality has been identified to affect other infant feeding and growth relationships. For example, earlier age at introduction of solid foods has been widely associated with larger infant body size and higher obesity risk, and therefore has been proposed as an early life intervention target for obesity prevention. However, we recently reported that, in most studies that collected data on infant growth before the age window for solid food introduction, rapid weight gain preceded the earlier introduction of solids, rather than vice versa.^12^ These infant feeding patterns (FF vs EBF; early vs later solids) are difficult to randomly allocate to in trials, and therefore the evidence relies largely on observational cohorts. The frequent changes and wide variation in feeding and growth during the first 6 months of life demand frequent study assessments in order to correctly assess the time sequence of changes in feeding and growth. Other studies have reported the demand for frequent feeding as a reason for stopping breast feeding but growth measurements prior to that change of feeding were unavailable.^13^ We are unaware of cohorts other than CBGS-BF that have collected sufficiently detailed information to address the current question.

We acknowledge that our study has a number of limitations. The sample size was small compared with other birth cohorts, which reflects a trade-off between study numbers and frequency and nature of assessments. Stringent inclusion and exclusion criteria were applied, in particular the exclusion of small babies and mothers with obesity, and the reliance of a single site with predominant white Caucasian population might limit the applicability of the results to more diverse populations. The exclusion of infant-mother pairs who did not receive EBF for at least 2 weeks has advantages and disadvantages. While this focused study sample minimised the confounding effects of many environmental factors, it limited the number of subjects eligible for inclusion.

These findings have a number of potential implications. Even among this highly educated study population who intended to EBF, only a minority (28/150; 18%) met the WHO and UNICEF recommendation to EBF for the first 6 months of life (26 or more weeks). We speculate that slower infant weight gains might have influenced some parents’ decisions to stop EBF before age 6 or 12 weeks. In the UK Infant Feeding Survey, ‘worry over not having enough milk’ was a major reason for stopping breast feeding and the majority of mothers who stopped would have liked to breast feed for longer.^14^ Furthermore, we conjecture that the optimal growth patterns described by the WHO Growth Standards might be inflated by selection bias in the underlying WHO Multicentre Growth Reference Study. The inclusion criteria for that study required infants to be exclusively or predominantly breast fed for at least 4 months. Of the 1737 infants who met the baseline health and sociodemographic criteria, only half (n=882) fulfilled the infant feeding and no-smoking criteria to be included in the longitudinal growth sample.^5^ Hence, it is possible that infants who grew initially faster on breast feeding were more likely to remain eligible and, possibly also due to the selection of high socioeconomic class families, might explain their early infancy size advantage when compared with other cohorts.^7 8^ In support of this interpretation, we note that the UK chose to combine the WHO Growth Standard (from age 2 weeks onwards) with the British 1990 reference at birth because the implied rate of weight gain according to WHO was deemed to be excessive during the first 2 weeks.^8^

In conclusion, in this cohort of initially EBF infants with frequent assessments of feeding and growth, we identified a ‘reverse’ relationship between slower initial weight gain and subsequent early introduction of FF. While we recognise there may be many reasons why infants stop EBF, we conjecture that broader recognition of the wide range of normal infant growth might encourage some parents to not stop EBF earlier than they intended.

## Data Availability

Data are available on reasonable request. Data are available on reasonable request to the Department of Paediatrics, University of Cambridge (https://paediatrics.medschl.cam.ac.uk/research/clinical-trials/cambridge-baby-growth-study/).
