# Supplementary material for: Early weight gain influences duration of breast feeding: prospective cohort study
Source: Arch Dis Child. 2022 Jul 15;107(11):1034–7. doi: 10.1136/archdischild-2022-323999 (PMC9606501; doi:10.1136/archdischild-2022-323999)
Supplement: Supplementary data [file archdischild-2022-323999supp001.pdf]

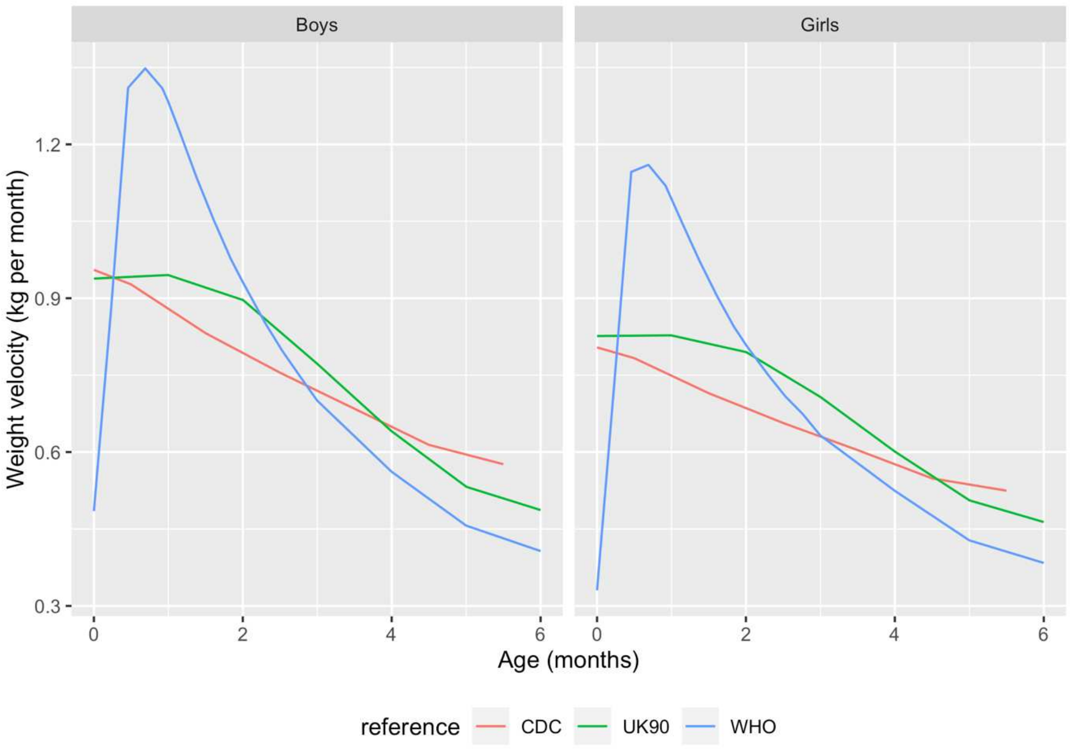

Supplementary Figure 1 – Early infant weight velocity plotted by age and stratified by sex, derived by differentiating the 50th centiles for weight indicated by the British 1990 (UK90), US Centers for Disease Control (CDC) or WHO International Growth Standard (WHO) growth references.

This figure was drawn at the suggestion of and using R code provided by Professor Tim Cole.
