# Supplementary material for: Early weight gain influences duration of breast feeding: prospective cohort study
Source: Arch Dis Child. 2022 Jul 15;107(11):1034–7. doi: 10.1136/archdischild-2022-323999 (PMC9606501; doi:10.1136/archdischild-2022-323999)
Supplement: Supplementary data [file archdischild-2022-323999supp002.pdf]

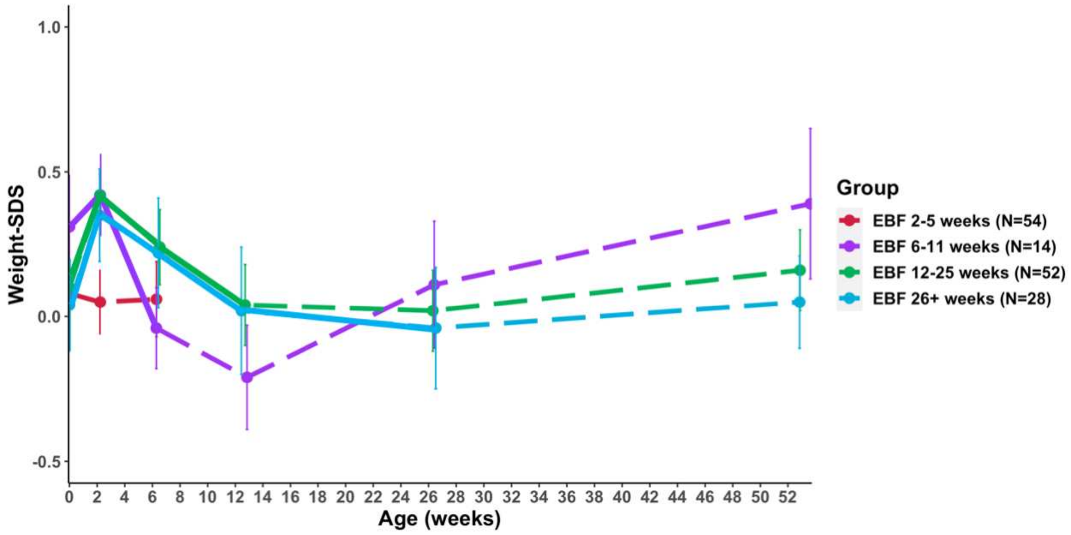

Supplementary Figure 2 - Infant weight standard deviation scores (SDS) from birth to 52 weeks, stratified by exclusive breastfeeding (EBF) duration (4 groups). Points and error bars indicate means and standard errors. The solid lines indicate the EBF period in each group.
